# Valvular Heart Disease-Related Mortality Between Middle- and High-Income Countries During 2000 to 2019

**DOI:** 10.1016/j.jacadv.2024.101133

**Published:** 2024-08-28

**Authors:** Makoto Hibino, Hiroki A. Ueyama, Michael E. Halkos, Kendra J. Grubb, Raj Verma, Azeem Majeed, Christoph A. Nienaber, Bobby Yanagawa, Deepak L. Bhatt, Subodh Verma

**Affiliations:** a Department of Thoracic and Cardiovascular Surgery, Heart, Vascular, and Thoracic Institute, Cleveland Clinic Cleveland, Ohio, USA; b Division of Cardiac Surgery, St. Michael's Hospital of Unity Health Toronto, Toronto, Ontario, Canada; c Division of Cardiology, Emory University School of Medicine, Atlanta, Georgia, USA; d Division of Cardiothoracic Surgery, Emory University School of Medicine, Atlanta, Georgia, USA; e Royal College of Surgeon in Ireland, Dublin, Ireland; f Department of Primary Care and Public Health, School of Public Health, Imperial College London, London, United Kingdom; g Cardiology and Aortic Centre, Royal Brompton and Harefield Hospitals, Guy's and St. Thomas' NHS Foundation Trust, London, United Kingdom; h National Heart and Lung Institute, Faculty of Medicine, Imperial College London, London, United Kingdom; i Department of Surgery, University of Toronto, Toronto, Ontario, Canada; j Mount Sinai Heart, Icahn School of Medicine at Mount Sinai, New York, New York, USA

**Keywords:** valvular heart disease, global mortality trends, national income levels

## Abstract

**Background:**

Valvular heart disease (VHD) management has evolved rapidly in recent decades, but disparities in health care access persist among countries with varying socioeconomic backgrounds.

**Objectives:**

The purpose of this study was to investigate global mortality trends from VHD and assess the difference between middle- and high-income countries.

**Methods:**

We obtained mortality data from the World Health Organization Mortality Database for VHD and its subgroups (rheumatic valvular disease [RVD], infective endocarditis [IE], aortic stenosis [AS], and mitral regurgitation [MR]) from 2000 to 2019. Age-specific and age-standardized mortality rates per 100,000 persons in middle- and high-income countries were calculated, and trends were analyzed using joinpoint regression.

**Results:**

A total of 93 countries (42 middle-income and 51 high-income) were included in the analysis. Both middle- and high-income countries showed an increasing trend in crude VHD mortality rate. In middle-income countries, the age-standardized VHD-related mortality rate was constant (0.0%/year), with decreasing RVD (−2.7%/year) and increasing IE, AS, and MR (0.8%/year, 2.0%/year, and 2.2%/year, respectively). In high-income countries, the age-standardized VHD-related mortality rate was decreasing (−0.6%/year). However, there was a rapid increase in mortality rate from IE in age ≤39 years after 2009 (7.0%/year). Moreover, there was a decreasing mortality rate from AS after 2015 but an increasing rate from MR after 2013, particularly in age ≥80 years.

**Conclusions:**

Our study identified a rising burden of VHD-related mortality worldwide. The distribution and trends of VHD mortality differed between middle- and high-income countries. Further investigation is needed to understand the underlying etiology of these varying mortality trends in VHD and its subgroups.

Valvular heart disease (VHD) is a significant cause of morbidity worldwide, with an estimated 74 million people affected in 2019.^1^^,^^2^ VHD ranks among the highest contributors to global deaths from cardiovascular disease.^3^ The epidemiology of VHD and its phenotypes is closely intertwined with geographical, demographical, and socioeconomic factors, leading to substantial global heterogeneity in its burden. Rheumatic valvular disease (RVD) remains the most common type of VHD worldwide, primarily impacting middle- and low-income countries.^1^ In contrast, high-income countries predominantly experience degenerative or functional valve disease, reflecting their aging populations.^1^

The management of VHD has rapidly evolved in recent decades. Improved access to health care, timely diagnosis, and antibiotic treatment have led to a substantial decrease in the burden of RVD in industrialized countries.^4^, ^5^, ^6^ Additionally, the development of transcatheter valve interventions, such as transcatheter aortic valve replacement and transcatheter edge-to-edge repair, has changed the landscape of treatment options for degenerative and functional valve disease, particularly benefiting elderly patients with a high comorbidity burden who were previously considered unsuitable for surgical intervention. As a result, there is a decreasing trend in mortality, especially in elderly patients with aortic stenosis (AS) in high-income countries.^7^^,^^8^ However, access to fundamental and advanced health care is characterized by substantial disparities among countries with varying socioeconomic backgrounds, causing inequity in both diagnosis and treatment. The impact of these disparities on mortality in VHD in the current era, as well as its trend in recent years, remains unclear. Understanding these trends is vital in developing effective global health care policies tailored to specific country needs.

The aim of this study was to investigate the global trends in mortality from VHD and determine the impact of socioeconomic backgrounds. We hypothesize that mortality from VHD is higher in middle-income countries and has improved in both middle-income and high-income countries.

## Material and methods

### Data source

The World Health Organization (WHO) Mortality Database^9^ receives official national statistics directly from the competent authorities of the contributing countries. Deaths in the database are recorded with an underlying cause of death, defined as the disease that initiated the events leading directly to death, according to the International Classification of Diseases (ICD). This database has been widely used for epidemiological research on a variety of diseases, including cardiothoracic diseases.^10^^,^^11^ We identified trends in mortality from VHD from 2000 to 2019. ICD-10 codes were used to determine the cause of death (VHD, from I05 to I08, from I33 to I38, and B376; RVD, from I05 to I08; infective endocarditis [IE], I33, I38, and B376; AS, I350; mitral regurgitation [MR], I340 and I341). Data on the age distribution of the countries’ populations at midyear were obtained from the United Nations World Population Prospects 2022.^12^ Country income levels, as determined by the World Bank based on gross national income per capita in 2019 were classified as follows: low income (≤$1,035 USD), middle income ($1,036-$12,535 USD), and high income (>$12,535 USD).^13^ Ethics approval was deemed unnecessary as all the data were publicly available, anonymous, and aggregated without any personal information.

### Data analysis

Crude mortality rates were calculated by dividing the number of deaths from each cause by the number of persons in a group of countries. Age-specific mortality rates were calculated by dividing the number of deaths by the number of persons in each age group (≤39, 40-64, 65-74, 75-84, ≥85 years). Using the WHO World Standard Population,^14^ age-standardized mortality rates in a group of countries were estimated with 95% CIs by using formulas developed by Tiwari.^15^ We included countries that reported mortality data for more than 9 years during the observation periods. We conducted joinpoint regression analysis to assess trends of age-specific and age-standardized mortality rates by estimating average annual percentage change (APC).^16^ Monte Carlo permutation method with 4499 randomly permuted data sets was used to identify an optimal joinpoint model with its 95% CI. The average APC and 95% CI were computed as a weighted average of APC from the joinpoint model. The trend was considered significantly increasing or decreasing when both limits of the 95% CI of an average APC were both positive and negative. Years with missing data were excluded from the analysis. Statistical analyses were performed using the STATA 17 statistical software (StataCorp LP) and Joinpoint Regression Program (Statistical Research and Applications Branch, National Cancer Institute). A *P* value <0.05 was considered statistically significant.

## Results

### Population

**Figure 1:**
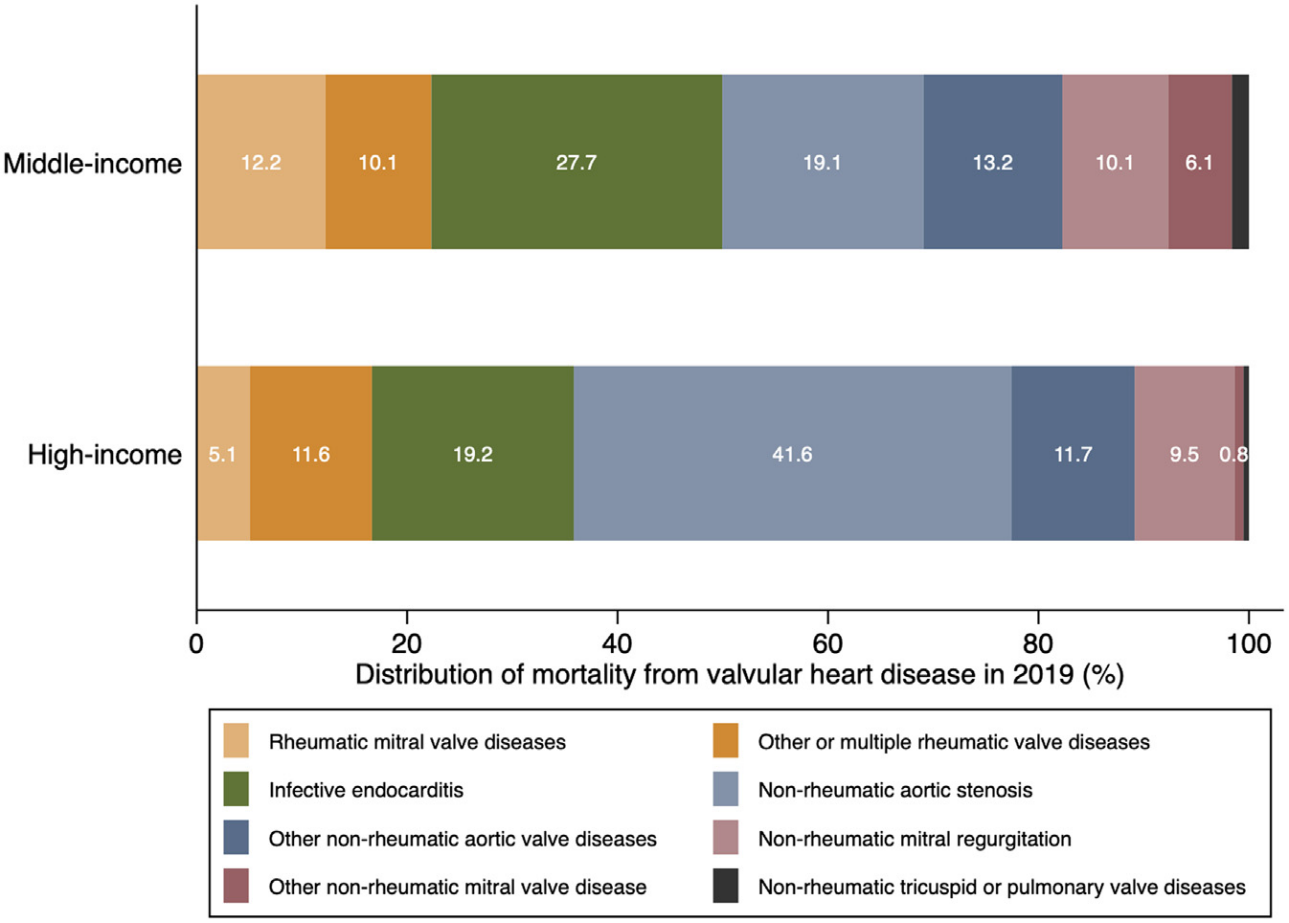
Distribution of Mortality From Valvular Heart Disease in 2019 in Middle- and High-Income Countries

Mortality data from 93 countries (42 middle-income and 51 high-income) between 2000 and 2019 were included in the study (Supplemental Tables 1 and 2). There were no low-income countries that met the inclusion criteria. Middle-income countries were primarily located in Central America, South America, Africa, Central Asia, Western Asia, and Southeastern Asia, whereas high-income countries were concentrated in North America, Europe, Eastern Asia, and Australia (Central Illustration). The average populations for middle-income countries and high-income countries during the observation periods were 943 million and 1,059 million, respectively, with an average observation period of 18.17 ± 3.05 years and 19.34 ± 2.57 years. The distribution of mortality from VHD differed between middle-income and high-income countries in 2019. Middle-income countries had a higher proportion of RVD (22.3% versus 16.7%) and IE (27.7% versus 19.2%), whereas high-income countries had a higher proportion of non-rheumatic AS, 41.6% versus 19.1% (Figure 1).

### Crude and age-standardized mortality rates stratified by income level

In 2019, the crude and age-standardized mortality rates of VHD in middle-income countries were 2.40 deaths per 100,000 and 2.39 deaths per 100,000 (95% CI: 2.36-2.42), and in high-income countries 11.16 deaths per 100,000 and 3.95 deaths per 100,000 (95% CI: 3.92-3.97) (Table 1). The higher mortality rate in high-income countries was predominantly from mortality related to AS.

**Table 1:** Crude and Age-Standardized Mortality Rate of Valvular Heart Disease and Its Subgroups

**Figure 2:**
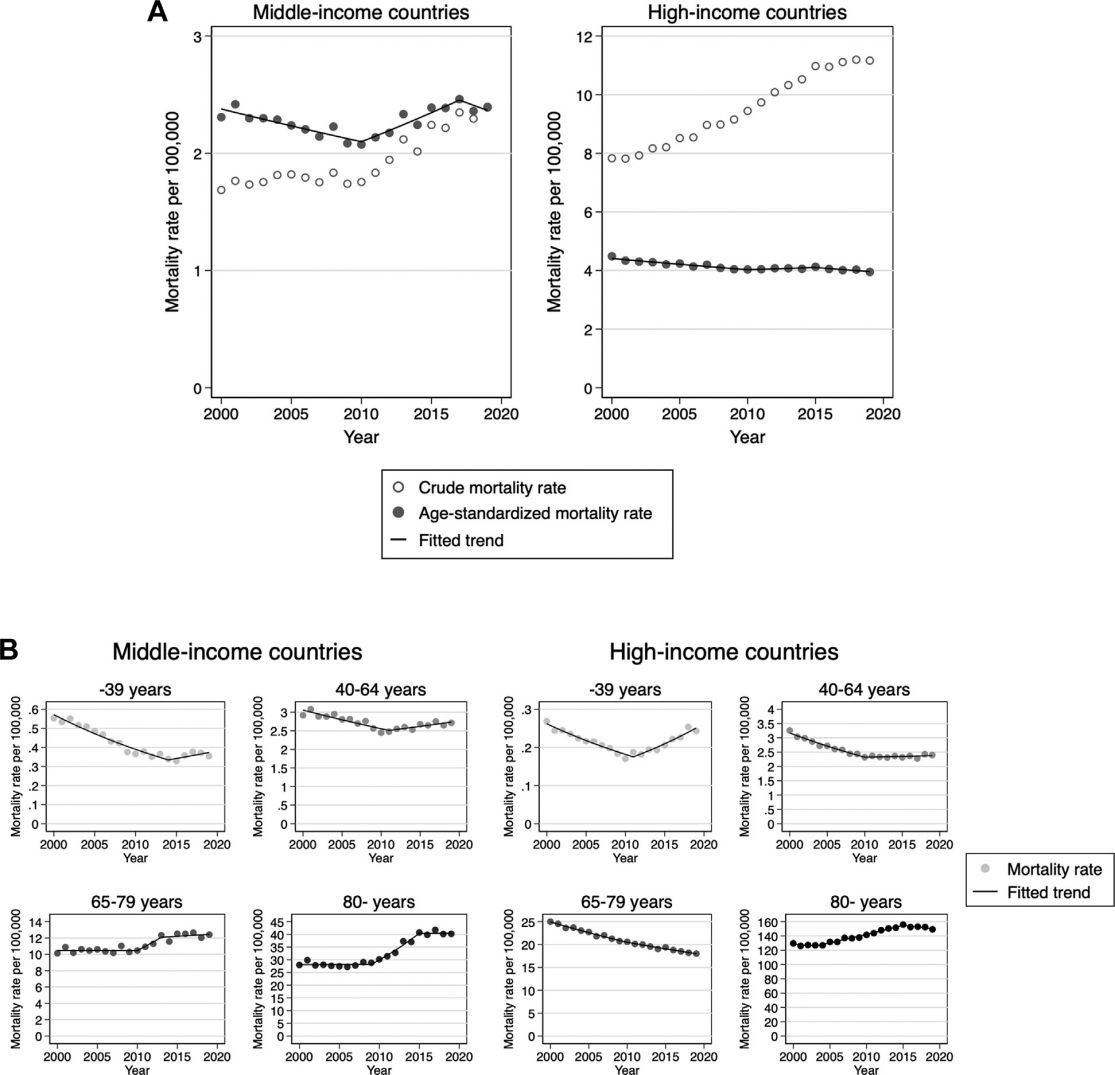
Crude, Age-Standardized, and Age-Specific Mortality Rates Per 100,000 from Valvular Heart Disease Stratified by Income Levels (A) Crude and age-standardized mortality rates per 100,000 from valvular heart disease stratified by income levels. (B) Age-specific mortality rates per 100,000 from valvular heart disease stratified by income levels.

**Figure 3:**
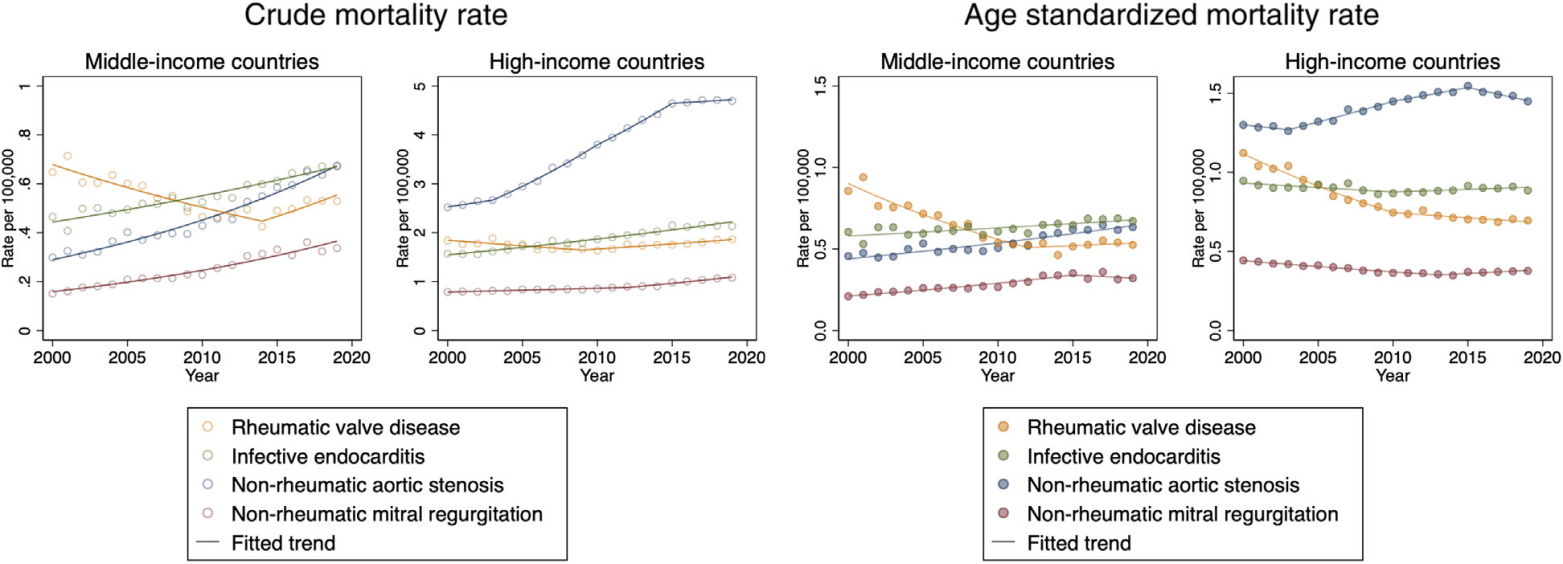
Crude and Age-Standardized Mortality Rates per 100,000 From the Subgroups of Valvular Heart Disease Stratified by Income Levels

In the crude population, both middle- and high-income countries experienced an increasing trend in VHD-related mortality rate and in subgroups IE, AS, and MR. (Table 1, Figure 2A). The RVD-related crude mortality rate showed a decreasing trend in middle-income countries (average APC of crude mortality rate: −1.1%/year, *P* = 0.031) and a constant trend in high-income countries (0.0%/year, *P* = 0.85) (Table 1, Supplemental Table 3, Figure 3).

For middle-income countries, the age-standardized VHD-related mortality rate was overall constant during the study period (Table 1, Figure 2A). However, it was characterized by a decreasing trend of RVD-related mortality rate (−2.7%/year, *P* < 0.001), and an increasing trend of mortality rates related to IE, AS, and MR (0.8%/year [*P* < 0.001], 2.0%/year [*P* < 0.001], and 2.2%/year [*P* < 0.001], respectively) (Table 1, Figure 3). The RVD-related mortality rate significantly decreased between 2000 and 2012 (−4.7%/year, *P* < 0.001) and remained constant thereafter (Table 2, Figure 3). Moreover, the mortality rates related to IE and AS showed a steady increase throughout the study period of 2000 to 2019 (IE: 0.8%/year, *P* < 0.001; AS: 2.0%/year, *P* < 0.001), and MR increased between 2000 and 2015 (3.2%/year, *P* < 0.001) and reached a plateau thereafter.

**Table 2:** Trend Change in Age-Standardized Mortality Rate of Valvular Heart Disease and Its Subgroups

In high-income countries, the age-standardized VHD-related mortality rate trend was decreasing during the study period (−0.6%/year, *P* < 0.001) (Table 1, Figure 2A). This trend was characterized by decreasing mortality rates related to RVD (−2.5%/year, *P* < 0.001) and MR (−0.8%/year, *P* < 0.001), increasing AS (0.6%/year, *P* = 0.005), and a constant trend for IE (−0.2%/year, *P* = 0.32) (Table 1, Figure 3). The RVD-related mortality rate decreased throughout the study period of 2000 to 2019 with a hinge point at 2010 (2000-2010: −4.0%/year, *P* < 0.001; 2010-2019: −0.9%/year, *P* = 0.008) (Table 2, Figure 3). Additionally, the mortality rate related to AS showed an increasing trend between 2003 and 2015 (2003-2010: 1.9%/year, *P* < 0.001; 2010-2015: 1.2%/year, *P* = 0.015), but a decreasing trend between 2015 and 2019 (−1.4%/year, *P* = 0.005). Conversely, the mortality rate related to MR decreased between 2000 and 2013 (−1.7%/year, *P* < 0.001) but shifted to an increasing trend between 2013 and 2019 (1.3%/year, *P* = 0.002).

### Age-specific mortality rates stratified by income level

The age-specific mortality rates from VHD and subgroups are summarized in Table 3 and Figure 2B. Overall, age-specific mortality rates from VHD and subgroups were higher in older age groups in both middle- and high-income countries. In younger age groups (≤39 years and 40-64 years), the age-specific mortality rates were generally similar between middle- and high-income countries, whereas in older age groups (65-79 years and ≥80 years), high-income countries had a higher age-specific mortality rate than middle-income countries.

**Table 3:** Age-Specific Mortality Rate of Valvular Heart Disease and Its Subgroups by 4 Age Groups

In middle-income countries, the age-specific mortality rate from overall VHD decreased in age groups ≤39 years (−2.2%/year, *P* < 0.001) and 40 to 64 years (−0.6%/year, *P* = 0.009), was constant in 65 to 79 years (0.9%/year, *P* = 0.22), and increased in ≥80 years (1.9%/year, *P* < 0.001) (Table 3, Figure 2B). The increasing trend in ≥80 years age group reached a plateau after 2015 (Supplemental Table 4, Figure 2B). The decrease in mortality rate in the age group ≤39 years was characterized by a decrease in mortality related to RVD and IE (Table 3, Supplemental Figures 1 and 2). In the age groups 40 to 64 years, 65 to 79 years, and ≥80 years, the mortality rate related to RVD decreased, but IE, AS, and MR increased (Table 3, Supplemental Table 5, Supplemental Figures 1-4). Specifically, in the age group ≥80 years, the increasing trend in the mortality rate from AS and MR reached a plateau after 2015 (Supplemental Table 5, Supplemental Figures 3 and 4).

For high-income countries, the age-specific mortality rate from overall VHD was constant in age group ≤39 years (−0.2%/year, *P* = 0.37), decreased in 40 to 64 years (−1.5%/year, *P* < 0.001) and 65 to 79 years (−1.7%/year, *P* < 0.001), and increased in ≥80 years (0.8%/year, *P* < 0.001) (Table 3, Figure 2B). The increase in mortality rate in the age group ≥80 years plateaued after 2015 (Supplemental Table 4, Figure 2B). In the age group ≤39 years, mortality related to RVD, AS, and MR decreased, but IE increased, particularly between 2009 to 2019 (7.0%/year, *P* < 0.001) (Supplemental Table 5, Supplemental Figures 1 to 4). In the age groups 40 to 64 years and 65 to 79 years, mortality rates from all subsets of VHD showed a general decreasing trend (Table 3 and Supplemental Figures 1-4). However, in the age group ≥80 years, the mortality rate from AS increased overall (1.4%/year, *P* < 0.001), characterized by an increase between 2004 and 2015 and a decrease thereafter (−1.1%/year, *P* = 0.011) (Table 3, Supplemental Table 5, Supplemental Figure 3). Additionally, the mortality rate in the age group ≥80 years from MR also exhibited an overall increase (0.5%/year, *P* = 0.007), particularly from 2013 to 2019 (2.2%/year, *P* < 0.001) (Table 3, Supplemental Table 5, Supplemental Figure 4).

## Discussion

This study investigating the global trend of mortality from VHD from 2000 to 2019 using the WHO Mortality Database has several important findings (Central Illustration). First, both middle- and high-income countries are experiencing an increase in the crude mortality rate of VHD, except for RVD which is decreasing in middle-income countries and remains constant in high-income countries. Of note, high-income countries had a higher crude and age-standardized mortality rate from VHD, primarily driven by AS. Second, in middle-income countries, the age-standardized VHD-related mortality rate has remained stable from 2000 to 2019, characterized by the following observations: 1) a shift in the cause of mortality from RVD to IE, AS, and MR; 2) this shift is mainly observed in the higher age groups; and 3) the increasing mortality rate related to AS and MR plateaued after 2015 in the age group ≥80 years. Third, in high-income countries, the age-standardized VHD-related mortality rate is decreasing, and the following patterns are noted: 1) a constant decrease in RVD-related mortality rate; 2) an increasing trend in mortality rate related to AS until 2015, followed by a decreasing trend thereafter, particularly in the age group ≥80 years; 3) a decreasing trend in mortality rate related to MR until 2013, but an increasing trend thereafter, particularly in the age group ≥80 years; and 4) an increasing mortality rate related to IE in the age group ≤39 years. This study expands on findings from investigations by the Global Burden of Disease Study Group^6^^,^^17^, ^18^, ^19^ through utilization of joinpoint regression analysis, thereby adding a novel dimension to our understanding of changes in trends of mortality within specific disease and age groups.

**Central Illustration:**
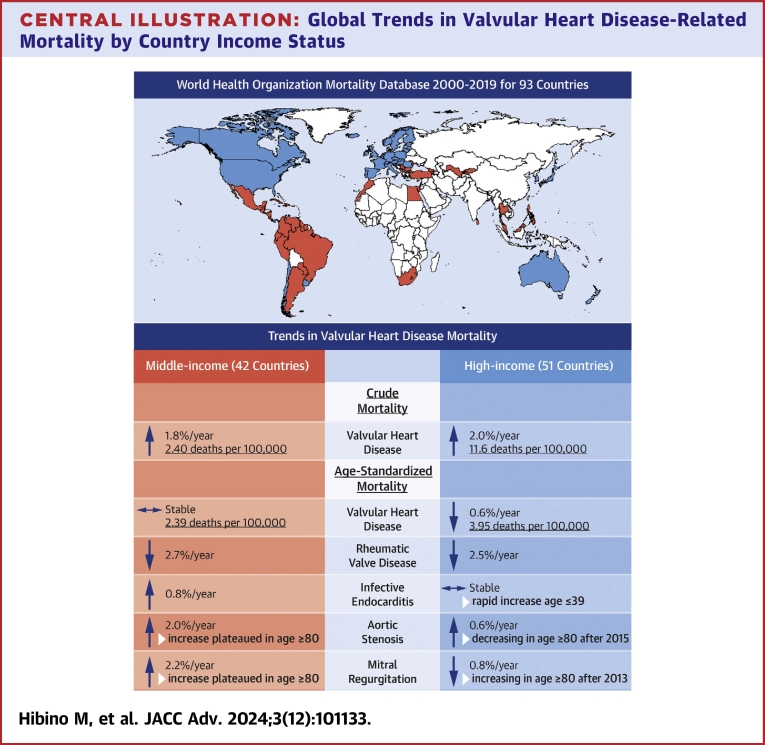
Global Trends in Valvular Heart Disease-Related Mortality by Country Income Status

Our study revealed distinct distributions of VHD between middle- and high-income countries. Middle-income countries exhibited a higher proportion of deaths from RVD and IE, while high-income countries showed a higher proportion of deaths from non-rheumatic AS. Interestingly, high-income countries exhibited higher age-standardized mortality from VHD compared to middle-income countries. The impact of national income level on VHD mortality and its distribution is multifactorial.^1^ First, there is a significant difference in the disease composition of VHD among countries. RVD displays geographical heterogeneity, concentrating in low- and middle-income countries due to strong associations with socioeconomic factors such as population density, poverty, and health care access.^20^^,^^21^ Conversely, high-income countries experience higher rates of degenerative and functional valve disease related to their aging populations.^17^ Additionally, the dissemination of advanced treatments, including surgical and transcatheter interventions, varies significantly between middle- and high-income countries.^22^, ^23^, ^24^, ^25^ Importantly, confounding factors impacting on VHD mortality should be considered. Resource-poor countries face limited access to diagnostic tools,^26^ likely resulting in significant underdiagnosis and underestimation of VHD-related mortality in such regions.^17^ Furthermore, disparities in health care are universal and can equally affect the diagnosis and treatment of other diseases, such as malignancies, which could act as competing risks of death, particularly in middle-income countries. This may lead to higher apparent mortality rates from VHD, especially among the elderly, such as AS, in high-income countries.^17^

In our study, both middle- and high-income countries revealed an increasing crude mortality rate from VHD, reflecting an aging population and highlighting the burden on health care, which is expected to grow further with aging. Middle-income countries maintained constant age-standardized VHD-related mortality rates, but the composition shifted from RVD to IE, AS, and MR, reflecting the successful implementation of control programs and health care improvements.^6^ The rising mortality trend in middle-income countries for IE, AS, and MR necessitates further investigation. This finding is potentially related to an aging population leading to increasing prevalence and increasing diagnosis due to better access to multimodality diagnostic tools,^2^^,^^27^ as evidenced by a notable rise primarily observed in older age groups within our study. Treatment of these conditions often involves surgical or transcatheter interventions, and policies to further expand their use may be needed to augment the trend. Notably, the dissemination of such procedures may have already influenced the most vulnerable age group, as indicated by a plateau in the increasing trend of mortality rates from AS and MR in those aged over 80 years after 2015. High-income countries experienced an overall decreasing trend in age-standardized VHD mortality, with continuing decreases in RVD and recent declines in AS mortality, likely due to the rapid expansion of transcatheter aortic valve replacement.^28^^,^^29^ However, the shift to a recent increase in mortality from MR requires further investigation, possibly linked to increasing diagnosis of MR coinciding with the development of transcatheter mitral interventions^30^ and growing awareness of mitral annular calcification-related mitral disease.^31^ Notably, we observed an alarming trend of rising mortality from IE in the age group ≤39, likely reflecting the opioid epidemic and associated injection drug use, warranting attention from policymakers.^32^^,^^33^

### Study Limitations

First, the WHO Mortality Database lacks individual- or population-level prevalence of comorbid conditions, restricting our ability to adjust for these factors. Second, detailed information on disease specifics, including prevalence and treatment for each VHD in this population, was unavailable. Therefore, the underlying mechanism of the observed trend remains theoretical and requires further research and our findings should be considered hypothesis-generating in nature. Third, this population-based analysis heavily relies on clinical diagnosis via ICD-10 coding—this approach is prone to misclassification, may reflect increased coding as a result of improved access to diagnostic modality or procedures (which can result in apparent, but not actual, increase in disease burden and mortality), and has been shown to result in a substantial underestimate of VHD as a principal or contributory cause of death when compared against postmortem analysis.^17^^,^^34^ Furthermore, we were unable to identify non-rheumatic multivalve disease as a cause of death due to the absence of specific ICD-10 codes for this condition. Lastly, the database does not contain data from low-income countries that meet the inclusion criteria. As the result, this study was confined to middle- and high-income countries. Despite these limitations, the value of WHO Mortality Database is widely appreciated in epidemiological research to provide the best contemporary country-level estimates of disease burden.^10^^,^^11^

## Conclusions

Our study from the WHO Mortality Database revealed a rising burden of VHD-related mortality worldwide. In middle-income countries, the age-adjusted mortality rate from VHD remained constant, accompanied by a shift from RVD to IE, AS, and MR. Conversely, in high-income countries, the age-standardized mortality rate from VHD decreased, with a recent decline in AS and a simultaneous increase in MR. Importantly, an alarming increase in IE was observed among individuals aged ≤39 years. Further investigation at the country level is necessary to understand the etiology behind these varying mortality trends in VHD and subgroups.

Perspectives **COMPETENCY IN SYSTEM-BASED PRACTICE:** Physicians and policymakers should be aware of the increasing burden of VHD-related mortality worldwide, with particular attention to specific trends in VHD and its subgroups in their respective countries based on income level.

**TRANSLATIONAL OUTLOOK:** To develop effective policies aimed at reducing mortality related to VHD, a comprehensive investigation into the detailed etiology behind the varying mortality trends in VHD and its subgroups is essential at the country level.

## Funding support and author disclosures

Dr Halkos is on the advisory board for Medtronic. Dr Grubb is on the advisory board for Medtronic, Boston Scientific, Abbott, and 4C Medical; and is a consultant for or received an honorarium from Medtronic, Boston Scientific, Abbott, and 4C Medical and Edwards Lifesciences. Dr Bhatt is on the advisory board for Angiowave, Bayer, Boehringer Ingelheim, CellProthera, Cereno Scientific, Elsevier Practice Update Cardiology, High Enroll, Janssen, Level Ex, McKinsey, Medscape Cardiology, Merck, MyoKardia, NirvaMed, Novo Nordisk, PhaseBio, PLx Pharma, Stasys; is on the Board of Directors for American Heart Association New York City, Angiowave (stock options), Bristol Myers Squibb (stock), DRS.LINQ (stock options), High Enroll (stock); is a consultant for Broadview Ventures, Hims, SFJ, Youngene; is on the Data Monitoring Committees for Acesion Pharma, Assistance Publique-Hôpitaux de Paris, Baim Institute for Clinical Research (formerly Harvard Clinical Research Institute, for the PORTICO trial, funded by 10.13039/100006279St. Jude Medical, now 10.13039/100000046Abbott), 10.13039/100008497Boston Scientific (Chair, PEITHO trial), Cleveland Clinic, Contego Medical (Chair, PERFORMANCE 2), 10.13039/100006513Duke Clinical Research Institute, 10.13039/100000871Mayo Clinic, Mount Sinai School of Medicine (for the ENVISAGE trial, funded by 10.13039/501100022274Daiichi Sankyo; for the ABILITY-DM trial, funded by 10.13039/100030895Concept Medical; for ALLAY-HF, funded by Alleviant Medical), 10.13039/100008272Novartis, 10.13039/100030936Population Health Research Institute; Rutgers University (for the 10.13039/100000002NIH-funded MINT Trial); has received honoraria from American College of Cardiology (Senior Associate Editor, Clinical Trials and News, ACC.org; Chair, ACC Accreditation Oversight Committee), Arnold and Porter law firm (work related to Sanofi/Bristol-Myers Squibb clopidogrel litigation), Baim Institute for Clinical Research (formerly Harvard Clinical Research Institute; RE-DUAL PCI clinical trial steering committee funded by 10.13039/100001003Boehringer Ingelheim; AEGIS-II executive committee funded by 10.13039/100008322CSL Behring), Belvoir Publications (Editor in Chief, Harvard Heart Letter), Canadian Medical and Surgical Knowledge Translation Research Group (clinical trial steering committees), CSL Behring (AHA lecture), Cowen and Company, 10.13039/100006513Duke Clinical Research Institute (clinical trial steering committees, including for the PRONOUNCE trial, funded by 10.13039/501100004914Ferring Pharmaceuticals), HMP Global (Editor in Chief, Journal of Invasive Cardiology), Journal of the American College of Cardiology (Guest Editor; Associate Editor), K2P (Co-Chair, interdisciplinary curriculum), Level Ex, Medtelligence/ReachMD (CME steering committees), MJH Life Sciences, Oakstone CME (Course Director, Comprehensive Review of Interventional Cardiology), Piper Sandler, Population Health Research Institute (for the COMPASS operations committee, publications committee, steering committee, and USA national co-leader, funded by 10.13039/100004326Bayer), WebMD (CME steering committees), Wiley (steering committee); other services from Clinical Cardiology (Deputy Editor); holds a patent on sotagliflozin (named on a patent for sotagliflozin assigned to Brigham and Women's Hospital who assigned to Lexicon; neither I nor Brigham and Women's Hospital receive any income from this patent); has received research funding from 10.13039/100000046Abbott, Acesion Pharma, Afimmune, Aker Biomarine, 10.13039/100006400Alnylam, Amarin, 10.13039/100002429Amgen, 10.13039/100004325AstraZeneca, 10.13039/100015340Bayer, Beren, 10.13039/100001003Boehringer Ingelheim, 10.13039/100008497Boston Scientific, 10.13039/100002491Bristol-Myers Squibb, Cardax, CellProthera, Cereno Scientific, 10.13039/100007560Chiesi, CinCor, Cleerly, 10.13039/100008322CSL Behring, 10.13039/501100023368Eisai, 10.13039/100009933Ethicon, Faraday Pharmaceuticals, 10.13039/501100004914Ferring Pharmaceuticals, 10.13039/100005632Forest Laboratories, Fractyl, Garmin, 10.13039/501100018679HLS Therapeutics, 10.13039/501100016198Idorsia, 10.13039/100010721Ironwood, Ischemix, 10.13039/100005565Janssen, Javelin, Lexicon, 10.13039/100004312Lilly, 10.13039/100004374Medtronic, 10.13039/100004334Merck, 10.13039/100019533Moderna, 10.13039/100016619MyoKardia, NirvaMed, 10.13039/100008272Novartis, 10.13039/501100004191Novo Nordisk, 10.13039/100019120Otsuka, Owkin, 10.13039/100004319Pfizer, PhaseBio, PLx Pharma, Recardio, 10.13039/100009857Regeneron, Reid Hoffman Foundation, 10.13039/100004337Roche, 10.13039/100004339Sanofi, Stasys, Synaptic, 10.13039/100015237The Medicines Company, Youngene, 89Bio; has received royalties from Elsevier (Editor, Braunwald’s Heart Disease); is a site co-investigator for Abbott, Biotronik, Boston Scientific, CSI, Endotronix, St. Jude Medical (now Abbott), Philips, SpectraWAVE, Svelte, Vascular Solutions; is a trustee for American College of Cardiology; and reports unfunded research from FlowCo. All other authors have reported that they have no relationships relevant to the contents of this paper to disclose.
