# Supplementary material for: Valvular Heart Disease-Related Mortality Between Middle- and High-Income Countries During 2000 to 2019
Source: JACC Adv. 2024 Aug 28;3(12):101133. doi: 10.1016/j.jacadv.2024.101133 (PMC11733992; doi:10.1016/j.jacadv.2024.101133)

**Supplemental Table 1.** Summary of countries included in analysis of valvular heart disease and its subgroups.

| **Category and subgroups of valve disease** | **Income category** | **Number of included countries** | **Average observation years per country (average ± SD)** | **Average total population in included countries per year** |
| --- | --- | --- | --- | --- |
| **Valvular heart disease** | Middle | 42 | 18.17 ± 3.05 | 943,180,035 |
|  | High | 51 | 19.34 ± 2.57 | 1,059,321,273 |
| Rheumatic valvular disease | Middle | 38 | 17.98 ± 3.56 | 938,846,742 |
|  | High | 45 | 19.34 ± 2.56 | 1,055,283,446 |
| Infective endocarditis | Middle | 37 | 18.04 ± 3.41 | 937,453,248 |
|  | High | 47 | 19.32 ± 2.67 | 1,056,684,428 |
| Aortic stenosis | Middle | 21 | 18.15 ± 3.39 | 571,787,344 |
|  | High | 41 | 19.20 ± 2.63 | 1,041,980,185 |
| Mitral regurgitation | Middle | 21 | 18.02 ± 3.67 | 571,199,305 |
|  | High | 39 | 18.97 ± 2.78 | 1,038,874,329 |

SD = standard deviation

**Supplemental Table 2.** List of countries included in analysis of valvular heart disease and its subgroups.

|  | **Middle-income countries** | **High-income countries** |
| --- | --- | --- |
| Valvular heart disease | Argentina, Armenia, Belize, Brazil, Bulgaria, Colombia, Costa Rica, Cuba, Dominica, Dominican Republic, Ecuador, Egypt, El Salvador, Fiji, Georgia, Grenada, Guatemala, Guyana, Jamaica, Kyrgyzstan, Malaysia, Maldives, Mauritius, Mexico, Montenegro, Morocco, Nicaragua, Paraguay, Peru, Philippines, Republic of Moldova, Saint Lucia, Saint Vincent and Grenadines, Serbia, South Africa, Sri Lanka, Suriname, TFYR Macedonia, Thailand, Turkey, Uzbekistan, Venezuela | Antigua and Barbuda, Aruba, Australia, Austria, Bahamas, Bahrain, Barbados, Belgium, Bermuda, Canada, Chile, Croatia, Cyprus, Czech Republic, Denmark, Estonia, Finland, France, Germany, Hong Kong SAR, Hungary, Iceland, Ireland, Israel, Italy, Japan, Kuwait, Latvia, Lithuania, Luxembourg, Malta, Netherlands, New Zealand, Norway, Panama, Poland, Portugal, Puerto Rico, Qatar, Republic of Korea, Romania, Slovakia, Slovenia, Spain, Sweden, Switzerland, Trinidad and Tobago, United Kingdom, United States of America, Uruguay, US Virgin Islands |
| Rheumatic valvular disease | Argentina, Armenia, Belize, Brazil, Bulgaria, Colombia, Costa Rica, Cuba, Dominican Republic, Ecuador, Egypt, El Salvador, Fiji, Georgia, Grenada, Guatemala, Guyana, Jamaica, Kyrgyzstan, Malaysia, Mauritius, Mexico, Morocco, Nicaragua, Paraguay, Peru, Philippines, Republic of Moldova, Saint Lucia, Serbia, South Africa, Sri Lanka, Suriname, TFYR Macedonia, Thailand, Turkey, Uzbekistan, Venezuela | Australia, Austria, Bahamas, Belgium, Canada, Chile, Croatia, Cyprus, Czech Republic, Denmark, Estonia, Finland, France, Germany, Hong Kong SAR, Hungary, Iceland, Ireland, Israel, Italy, Japan, Kuwait, Latvia, Lithuania, Luxembourg, Malta, Netherlands, New Zealand, Norway, Panama, Poland, Portugal, Puerto Rico, Qatar, Republic of Korea, Romania, Slovakia, Slovenia, Spain, Sweden, Switzerland, Trinidad and Tobago, United Kingdom, United States of America, Uruguay |
| Infective endocarditis | Argentina, Armenia, Brazil, Bulgaria, Colombia, Costa Rica, Cuba, Dominica, Dominican Republic, Ecuador, Egypt, El Salvador, Fiji, Georgia, Grenada, Guatemala, Guyana, Jamaica, Kyrgyzstan, Malaysia, Mauritius, Mexico, Morocco, Nicaragua, Paraguay, Peru, Philippines, Republic of Moldova, Saint Lucia, Serbia, South Africa, Sri Lanka, Suriname, Thailand, Turkey, Uzbekistan, Venezuela | Australia, Austria, Bahamas, Barbados, Belgium, Canada, Chile, Croatia, Cyprus, Czech Republic, Denmark, Estonia, Finland, France, Germany, Hong Kong SAR, Hungary, Iceland, Ireland, Israel, Italy, Japan, Kuwait, Latvia, Lithuania, Luxembourg, Malta, Netherlands, New Zealand, Norway, Panama, Poland, Portugal, Puerto Rico, Qatar, Republic of Korea, Romania, Slovakia, Slovenia, Spain, Sweden, Switzerland, Trinidad and Tobago, United Kingdom, United States of America, Uruguay, US Virgin Islands |
| Aortic stenosis | Argentina, Brazil, Colombia, Costa Rica, Cuba, Dominican Republic, Ecuador, El Salvador, Georgia, Guatemala, Guyana, Kyrgyzstan, Mauritius, Mexico, Morocco, Nicaragua, Paraguay, Peru, Republic of Moldova, Turkey, Venezuela | Australia, Austria, Belgium, Bermuda, Canada, Chile, Croatia, Cyprus, Czech Republic, Denmark, Estonia, France, Germany, Hong Kong SAR, Hungary, Iceland, Ireland, Israel, Italy, Japan, Kuwait, Latvia, Lithuania, Luxembourg, Malta, Netherlands, New Zealand, Norway, Panama, Poland, Portugal, Puerto Rico, Republic of Korea, Romania, Spain, Sweden, Switzerland, Trinidad and Tobago United Kingdom United States of America Uruguay |
| Mitral regurgitation | Argentina, Brazil, Colombia, Costa Rica, Cuba, Dominican Republic, Ecuador, Georgia, Guatemala, Guyana, Jamaica, Kyrgyzstan, Mexico, Morocco, Nicaragua, Paraguay, Peru, Republic of Moldova, Suriname, Turkey, Venezuela | Australia, Austria, Belgium, Canada, Chile, Croatia, Cyprus, Czech Republic, Denmark, Estonia, France, Germany, Hong Kong SAR, Hungary, Ireland, Israel, Italy, Japan, Kuwait, Latvia, Lithuania, Luxembourg, Malta, Netherlands, New Zealand, Norway, Panama, Poland, Portugal, Puerto Rico, Republic of Korea, Romania, Spain, Sweden, Switzerland, Trinidad and Tobago, United Kingdom, United States of America, Uruguay |

**Supplemental Table 3.** Trend change in crude mortality rate of valvular heart disease and its subgroups.

| **Category and subgroups of valve disease** | **Income category** | **From** | **To** | **Average Annual percentage change (95% CI)** | **P-Value** |
| --- | --- | --- | --- | --- | --- |
| **Valvular heart disease** | Middle | 2000 | 2010 | 0.4% (-0.4, 1.2) | 0.28 |
|  |  | 2010 | 2019 | 3.4% (2.6, 4.2) | <0.001 |
|  | High | 2000 | 2009 | 2.0% (1.7, 2.3) | <0.001 |
|  |  | 2009 | 2015 | 3.0% (2.3, 3.6) | <0.001 |
|  |  | 2015 | 2019 | 0.6% (-0.2, 1.5) | 0.13 |
| Rheumatic valvular disease | Middle | 2000 | 2014 | -2.9% (-3.6, -2.2) | <0.001 |
|  |  | 2014 | 2019 | 4.4% (0.9, 8.0) | 0.017 |
|  | High | 2000 | 2009 | -1.3% (-1.9, -0.6) | 0.001 |
|  |  | 2009 | 2019 | 1.2% (0.7, 1.8) | <0.001 |
| Infective endocarditis | Middle | 2000 | 2019 | 2.2% (1.9, 2.6) | <0.001 |
|  | High | 2000 | 2019 | 1.9% (1.7, 2.1) | <0.001 |
| Aortic stenosis | Middle | 2000 | 2019 | 4.5% (4.1, 5.0) | <0.001 |
|  | High | 2000 | 2003 | 1.8% (-0.2, 3.9) | 0.079 |
|  |  | 2003 | 2010 | 5.2% (4.6, 5.8) | <0.001 |
|  |  | 2010 | 2015 | 4.1%(3.2, 5.1) | <0.001 |
|  |  | 2015 | 2019 | 0.4% (-0.5, 1.3) | 0.34 |
| Mitral regurgitation | Middle | 2000 | 2019 | 4.5% (3.9, 5.0) | <0.001 |
|  | High | 2000 | 2013 | 0.9% (0.6, 1.2) | <0.001 |
|  |  | 2013 | 2019 | 3.2% (2.6, 3.8) | <0.001 |

**Supplemental Table 4.** Trend change in age-specific mortality rate of valvular heart disease by 4 age groups.

|  | **≤** 3**9 years** | | | **40-64 years** | | | **65-79 years** | | | **≥** **80 years** | | | |
| --- | --- | --- | --- | --- | --- | --- | --- | --- | --- | --- | --- | --- | --- |
| **Income category** | **Periods** | **Annual percentage change (95% CI)** | **P-Value** | **Periods** | **Annual percentage change (95% CI)** | **P-Value** | **Periods** | **Annual percentage change (95% CI)** | **P-Value** | **Periods** | **Annual percentage change (95% CI)** | **P-Value** |  |
| Middle | 2000-2014 | -3.8% (-4.3, -3.2) | <0.001 | 2000-2011 | -1.8% (-2.3, -1.2) | <0.001 | 2000-2010 | 0.0% (-0.9, 0.9) | 0.98 | 2000-2009 | 0.0% (-1.0, 1.1) | 0.93 |  |
|  | 2014-2019 | 2.2% (-0.9, 5.5) | 0.15 | 2011-2019 | 1.1% (0.2, 1.9) | 0.015 | 2010-2013 | 4.9% (-4.5, 15.3) | 0.29 | 2009-2015 | 6.2% (4.2, 8.2) | <0.001 |  |
|  |  |  |  |  |  |  | 2013-2019 | 0.5% (-0.9, 1.9) | 0.46 | 2015-2019 | 0.0% (-2.2, 2.3) | 0.98 |  |
| High | 2000-2011 | -3.6% (-4.2, -3.0) | <0.001 | 2000-2010 | -3.1% (-3.5, -2.7) | <0.001 | 2000-2010 | -2.0% (-2.2, -1.7) | <0.001 | 2000-2003 | -0.7% (-2.6, 1.3) | 0.45 |  |
|  | 2011-2019 | 4.6% (3.6, 5.6) | <0.001 | 2010-2019 | 0.3% (-0.3, 0.8) | 0.31 | 2010-2019 | -1.4% (-1.7, -1.2) | <0.001 | 2003-2015 | 1.7% (1.5, 2.0) | <0.001 |  |
|  |  |  |  |  |  |  |  |  |  | 2015-2019 | -0.8% (-1.6, 0.0) | 0.060 |  |

**Supplemental Table 5.** Trend change in age-specific mortality rate of subgroups of valvular heart disease by 4 age groups.

|  |  | **≤ 39 years** | | | **40-64 years** | | | **65-79 years** | | | **≥ 80 years** | | | |
| --- | --- | --- | --- | --- | --- | --- | --- | --- | --- | --- | --- | --- | --- | --- |
|  | **Income category** | **Periods** | **Annual percentage change (95% CI)** | **P-Value** | **Periods** | **Annual percentage change (95% CI)** | **P-Value** | **Periods** | **Annual percentage change (95% CI)** | **P-Value** | **Periods** | **Annual percentage change (95% CI)** | **P-Value** |  |
| Rheumatic valvular disease | Middle | 2000-2004 | -2.1% (-7.3, 3.3) | 0.39 | 2000-2013 | -4.7% (-5.6, -3.8) | <0.001 | 2000-2019 | -2.0% (-2.6, -1.3) | <0.001 | 2000-2004 | -10.1% (-17.3, -2.3) | 0.016 |  |
|  |  | 2004-2009 | -11.5% (-16.9, -5.8) | 0.002 | 2013-2019 | 1.5% (-1.4, 4.6) | 0.29 |  |  |  | 2004-2019 | 0.7% (-0.3, 1.6) | 0.16 |  |
|  |  | 2009-2015 | -3.2% (-8.0, 1.8) | 0.17 |  |  |  |  |  |  |  |  |  |  |
|  |  | 2015-2019 | 7.5% (0.2, 15.3) | 0.044 |  |  |  |  |  |  |  |  |  |  |
|  | High | 2000-2013 | -5.1% (-6.3, -3.9) | <0.001 | 2000-2008 | -7.5% (-8.3, -6.7) | <0.001 | 2000-2009 | -4.8% (-5.5, -4.2) | <0.001 | 2000-2009 | -0.5% (-1.3, 0.3) | 0.23 |  |
|  |  | 2013-2019 | 6.6% (1.8, 11.6) | 0.01 | 2008-2015 | -3.6% (-5.1, -2.1) | <0.001 | 2009-2019 | -2.7% (-3.3, -2.0) | <0.001 | 2009-2019 | 0.8% (0.2, 1.3) | 0.011 |  |
|  |  |  |  |  | 2015-2019 | 0.9% (-2.2, 4.0) | 0.56 |  |  |  |  |  |  |  |
| Infective endocarditis | Middle | 2000-2019 | -1.5% (-2.1, -0.9) | <0.001 | 2000-2019 | 0.2% (-0.1, 0.6) | 0.22 | 2000-2019 | 2.2% (1.8, 2.6) | <0.001 | 2000-2008 | 1.2% (-0.7, 3.1) | 0.20 |  |
|  |  |  |  |  |  |  |  |  |  |  | 2008-2014 | 6.3% (3.4, 9.2) | <0.001 |  |
|  |  |  |  |  |  |  |  |  |  |  | 2014-2019 | 1.2% (-1.0, 3.6) | 0.26 |  |
|  | High | 2000-2009 | -3.7% (-5.0, -2.3) | <0.001 | 2000-2011 | -1.0% (-1.6, -0.4) | 0.003 | 2000-2019 | -1.0% (-1.2, -0.8) | <0.001 | 2000-2015 | 1.1% (0.8, 1.4) | <0.001 |  |
|  |  | 2009-2019 | 7.0% (5.8, 8.2) | <0.001 | 2011-2019 | 1.2% (0.2, 2.2) | 0.017 |  |  |  | 2015-2019 | -1.8% (-3.5, -0.0) | 0.049 |  |
| Aortic stenosis | Middle | 2000-2019 | 0.2% (-0.7, 1.0) | 0.71 | 2000-2019 | 1.7% (1.0, 2.3) | <0.001 | 2000-2019 | 1.7% (1.2, 2.2) | <0.001 | 2000-2011 | 1.9% (0.9, 3.0) | 0.002 |  |
|  |  |  |  |  |  |  |  |  |  |  | 2011-2015 | 6.4% (0.8, 12.3) | 0.028 |  |
|  |  |  |  |  |  |  |  |  |  |  | 2015-2019 | -0.4% (-3.4, 2.7) | 0.77 |  |
|  | High | 2000-2003 | -12.8% (-24.3, 0.5) | 0.058 | 2000-2004 | -2.6% (-5.7, 0.6) | 0.10 | 2000-2005 | -1.3% (-2.7, 0.2) | 0.085 | 2000-2004 | -0.5% (-1.7, 0.8) | 0.40 |  |
|  |  | 2003-2019 | -1.8% (-3.1, -0.6) | 0.008 | 2004-2019 | 0.9% (0.5, 1.3) | <0.001 | 2005-2010 | 1.6% (-0.4, 3.6) | 0.10 | 2004-2007 | 4.3% (0.8, 7.9) | 0.020 |  |
|  |  |  |  |  |  |  |  | 2010-2019 | -1.0% (-1.5, -0.4) | 0.002 | 2007-2015 | 2.6% (2.2, 3.0) | <0.001 |  |
|  |  |  |  |  |  |  |  |  |  |  | 2015-2019 | -1.1% (-1.9, -0.3) | 0.011 |  |
| Mitral regurgitation | Middle | 2000-2019 | -0.3% (-1.0, 0.5) | 0.47 | 2000-2019 | 2.0% (1.3, 2.7) | <0.001 | 2000-2017 | 3.6% (2.9, 4.4) | <0.001 | 2000-2015 | 4.8% (4.1, 5.6) | <0.001 |  |
|  |  |  |  |  |  |  |  | 2017-2019 | -7.9% (-21.5, 8.0) | 0.29 | 2015-2019 | -0.9% (-4.6, 2.8) | 0.59 |  |
|  | High | 2000-2019 | -4.4% (-5.0, -3.8) | <0.001 | 2000-2011 | -2.6% (-3.3, -1.9) | <0.001 | 2000-2013 | -2.7% (-3.0, -2.3) | <0.001 | 2000-2013 | -0.3% (-0.7, 0.0) | 0.050 |  |
|  |  |  |  |  | 2011-2019 | 1.2% (0.0, 2.3) | 0.043 | 2013-2019 | 0.8% (-0.3, 1.8) | 0.16 | 2013-2019 | 2.2% (1.3, 3.2) | <0.001 |  |

**Supplemental Figure 1.** Age-specific mortality rates per 100,000 from rheumatic valve disease stratified by income levels.


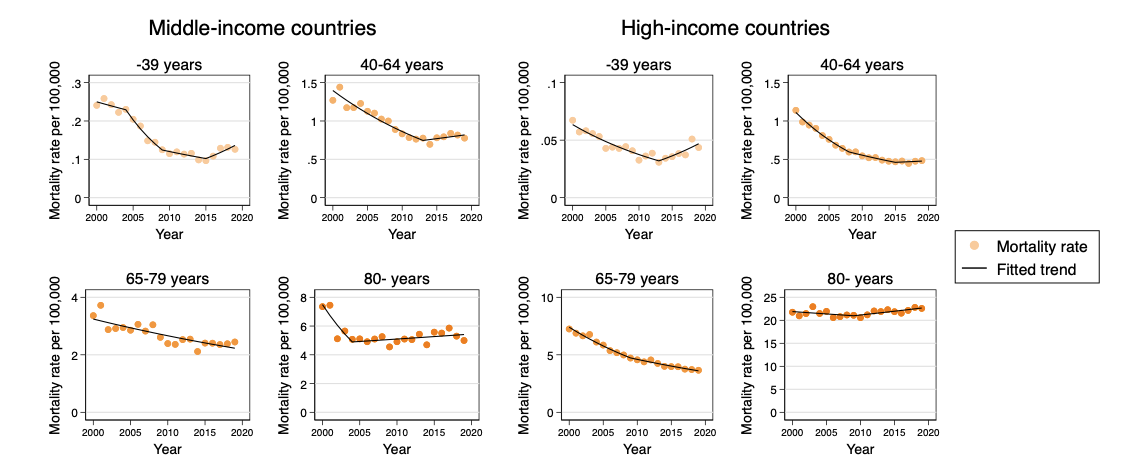


**Supplemental Figure 2.** Age-specific mortality rates per 100,000 from infective endocarditis stratified by income levels.

**
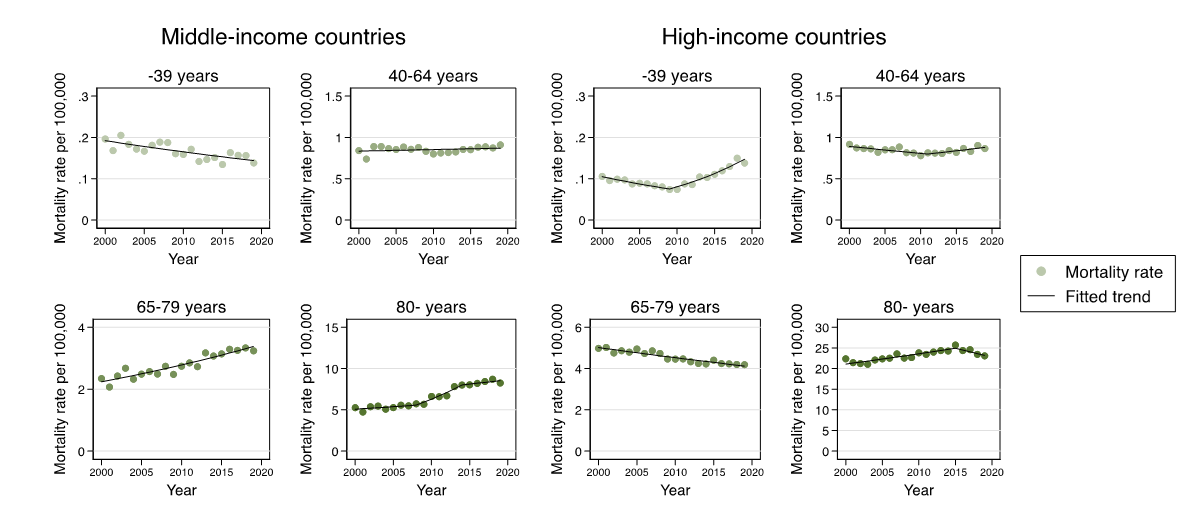
**

**Supplemental Figure 3.** Age-specific mortality rates per 100,000 from aortic stenosis stratified by income levels.

**
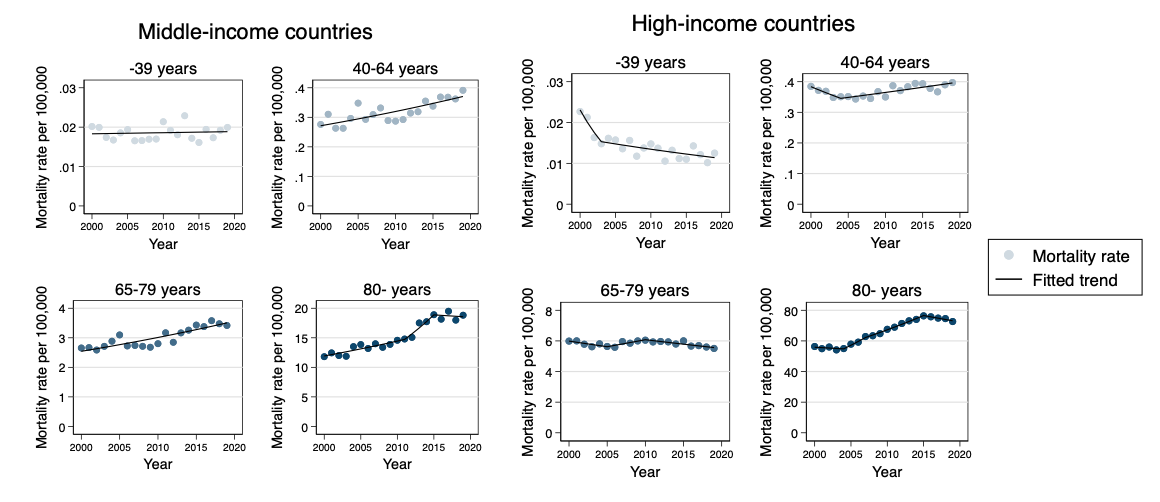
**

**Supplemental Figure 4.** Age-specific mortality rates per 100,000 from mitral regurgitation stratified by income levels.


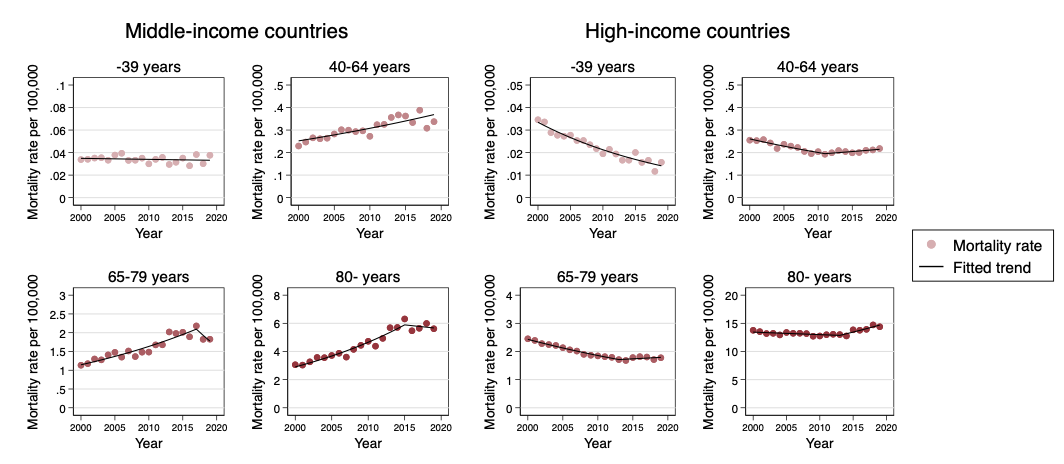

Supplement: Supplemental material [file mmc1.docx]
